# Advancements in Face and Neck Contouring: Integrating Radiofrequency-Assisted Liposuction with FaceTite and Buccal Fat Pad Excision for Facial Slimming

**DOI:** 10.1007/s00266-024-04245-1

**Published:** 2024-07-29

**Authors:** Matilde Tettamanzi, Giovanni Muratore, Giovanni Arrica, Roberto Cuomo, Edoardo Beatrici, Ilaria Ginatempo, Claudia Trignano, Corrado Rubino, Emilio Trignano

**Affiliations:** 1 Department of Surgical, Microsurgical and Medical Sciences, Plastic Surgery Unit, University of Sassari, Sassari, Italy; 2 Unit of Plastic and Reconstructive Surgery, Department of Medicine, Surgery and Neuroscience, University of Siena, Siena, Italy; 3 Department of Urology, Humanitas Research Hospital – IRCCS, Milan, Italy; 4 Department of Biomedical Sciences, University of Sassari, Sassari, Italy

**Keywords:** Neck contouring, FaceTite, Buccal fat pad, Facial skin tightening, Radiofrequency

## Abstract

**Background:**

The integration of neck liposuction with FaceTite Manipulus Radiofrequency (RF) technology and buccal fat pad excision for enhanced neck rejuvenation promise heightened precision and efficacy in sculpting the neck and jawline. Neck liposuction, coupled with RF technology, provides controlled thermal energy for adipose tissue treatment and collagen remodeling, while buccal fat pad excision offers refined contouring of the lower face and neck. This integrated approach aims to optimize patient outcomes and advance the field of esthetic plastic surgery.

**Methods:**

A prospective study was conducted from 2016 to 2023 on 80 consecutive patients who presented to the author's private clinic and required neck remodeling surgery for esthetic purposes. Patients were monitored and clinic appointments were scheduled at intervals of 0, 1, 3, 6 and 12 months post-treatment for evaluation. A tape measure recorded submental length at 1 and 6 months, and a satisfaction survey was administered one week before surgery and after six months. Physicians assessed improvement using a five-point scale for patient satisfaction and a four-point scale for overall improvement.

**Results:**

All patients underwent successful RFAL treatment, consistently achieving satisfaction with the outcomes. The average reduction in submental length measured 23 mm during the 6-month follow-up period. Additionally, the removal of buccal fat pads played a pivotal role in facial slimming and enhancing the esthetics of the upper cheek region.

**Conclusions:**

The integration of neck liposuction with FaceTite RF technology and buccal fat pad excision offers a promising approach for enhanced neck rejuvenation and facial contouring. This combined method demonstrates heightened precision and efficacy in sculpting the neck and jawline, aiming to optimize patient outcomes and advance the field of esthetic plastic surgery.

**Level of Evidence IV:**

This journal requires that authors assign a level of evidence to each article. For a full description of these Evidence-Based Medicine ratings, please refer to the Table of Contents or the online Instructions to Authors www.springer.com/00266.

## Introduction

The pursuit of a harmonious and youthful neck contour has been a perennial focus in the field of esthetic plastic surgery. As technological innovations continue to redefine the landscape of cosmetic procedures, the integration of advanced techniques becomes paramount in achieving optimal outcomes for patients seeking neck rejuvenation [1, 2]. This paper delves into the synergistic application of neck liposuction with radiofrequency (RF) technology using the FaceTite Manipulus, coupled with the nuanced approach of buccal fat pad excision. These combined modalities represent a tool in the evolution of neck sculpting, promising enhanced precision, efficacy, and patient satisfaction. In recent years, neck liposuction has gained widespread popularity as a minimally invasive solution for addressing submental fullness and enhancing the overall definition of the neck and jawline. The integration of RF technology, specifically with the FaceTite Manipulus, introduces a new dimension to this procedure by providing controlled thermal energy to target adipose tissue. This not only facilitates smoother fat extraction but also stimulates collagen remodeling, promoting skin contraction and tightening for a more comprehensive rejuvenation. Furthermore, the inclusion of buccal fat pad excision in the neck contouring repertoire adds a unique facet to the surgical approach [3]. The buccal fat pad, long recognized for its role in facial fullness, can significantly contribute to the appearance of an undefined neck and jawline. By strategically addressing this fat pad, surgeons can refine and sculpt the lower face and neck contours [4]. This paper aims to explore the scientific basis, procedural nuances, and clinical outcomes associated with the integration of neck liposuction, RF technology utilizing the FaceTite manipulus, and buccal fat pad excision. Through a comprehensive review of relevant literature, case studies, and emerging trends, we endeavor to provide a holistic understanding of the synergistic benefits that arise from the convergence of these advanced techniques in the realm of esthetic plastic surgery. We delve into the intricacies of this integrated approach to contribute valuable insights that can guide practitioners in optimizing patient outcomes and advancing the field of lower face and neck rejuvenation.

## Methods

**Fig. 1:**
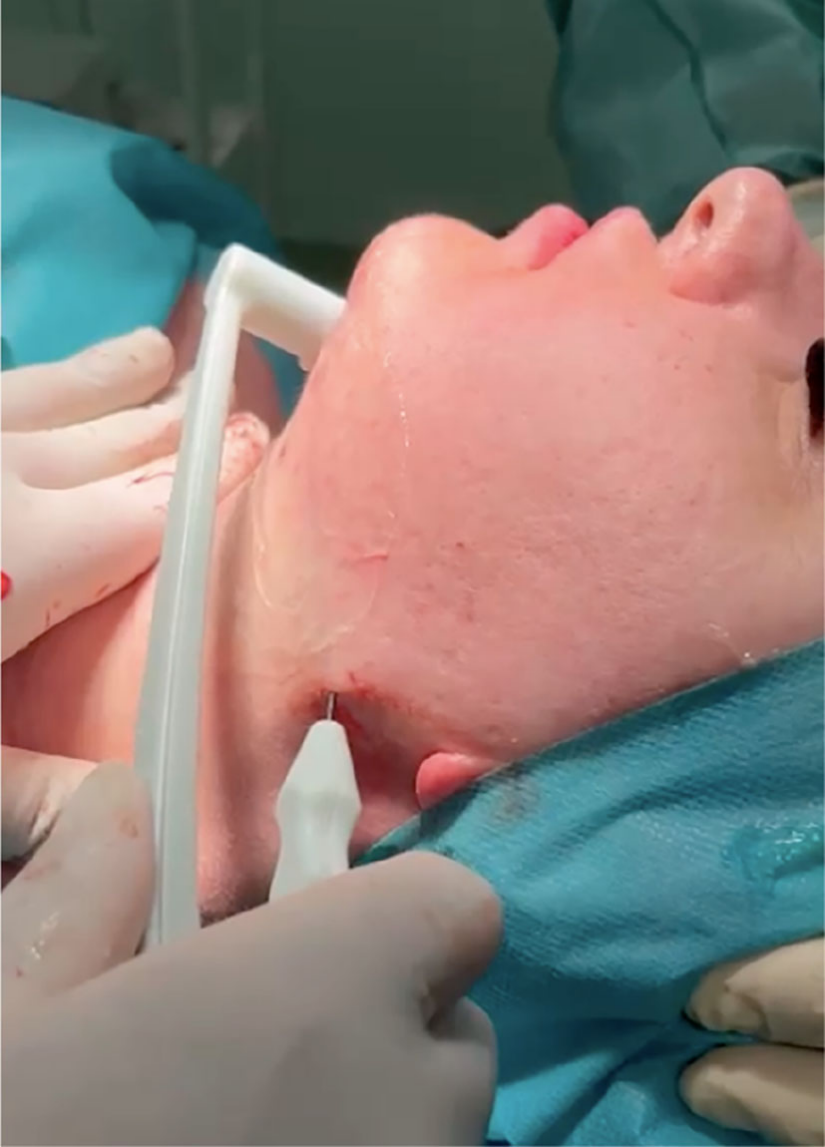
Neck tightening procedure with the FaceTite manipulus

**Fig. 2:**
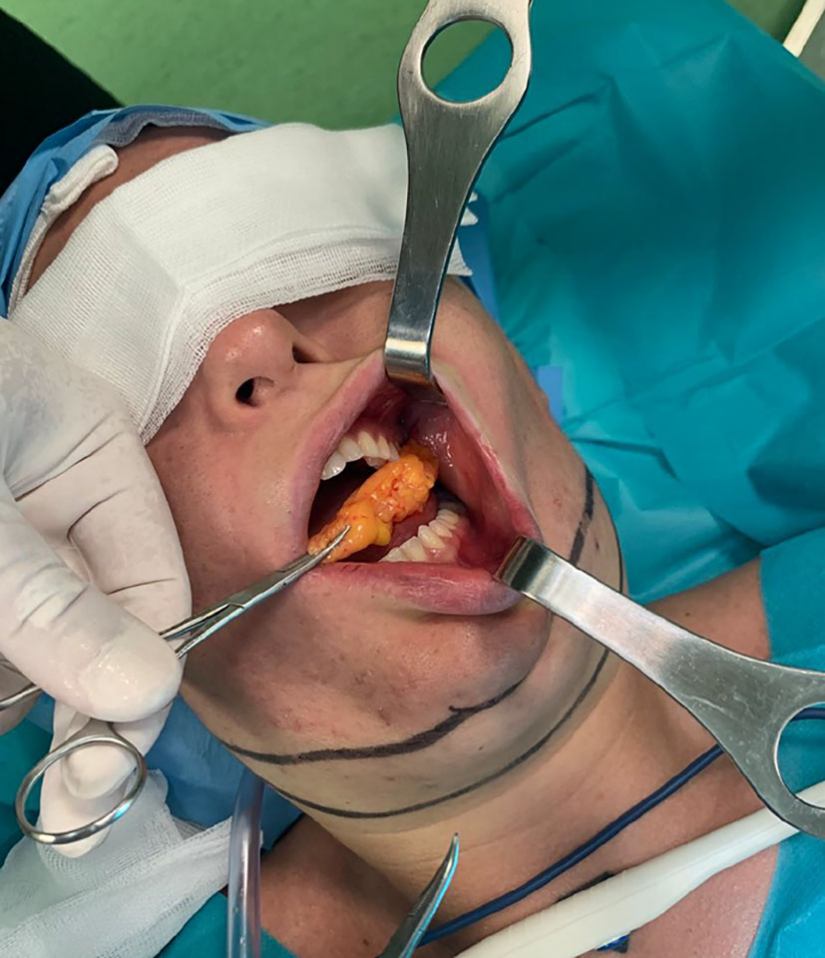
Buccal fat pad removal

From 2016 to 2023, 80 patients underwent neck remodeling with radio-frequency-assisted liposuction (RFAL). All procedures were performed in the author’s accredited outpatient clinic. The surgical team comprised a board-certified plastic surgeon, an assistant surgeon, an operating room nurse, and a board-certified anesthesiologist. All patients selected for the study were informed about the procedure and signed consent was obtained. Patients who needed improved neck contour were monitored and clinic appointments were scheduled at intervals of 1, 3, 6 and 12 months following the initial treatment sessions. Clinical evaluations were conducted at 1 month and then again at 6 months post-treatment. These appointments were designated as short-term and long-term follow-up assessments, respectively. A tape measure was used to record the submental length in a standing position at 1 and 6 months; the tape measure was kept in constant contact with the skin along the circumference from one earlobe to the other via the center of the chin [5]. One week before the surgery and after six months of follow-up, a survey was administered to each patient to assess the degree of satisfaction with the appearance of the neck and treatment satisfaction. The post-treatment result was compared with that before surgery to evaluate any improvement following treatment. A five-point scale was used: 0 indicated dissatisfaction, 1 signified slight satisfaction, 2 represented satisfaction, 3 indicated very high satisfaction, and 4 reflected extreme satisfaction. Physicians utilized a four-point scale to assess overall improvement: 0 for worsened condition, 1 for unchanged status, 2 for mild improvement, and 3 for significant improvement. Patients were treated in the neck using the FaceTite RFAL device. The FaceTite’s handpiece has two electrodes: an internal cannula emits simultaneous energy and suction in radio frequency (RF) and an external electrode reflects heat in the dermis. The RF energy causes a contraction of the fibrous septae surrounding the fat globules. When delivered at stratified depths, this RF energy tightens the connection of the skin and fat layer to the underlying fascia and the overlying dermis. This methodology allows the improvement of skin laxity in the neck and mandible region. FaceTite also delivers double-sided skin heating. The handpiece features a unique external thermistor directly above the internal RF cannula tip; so directional heating occurs only between the cannula tip and the external electrode, which minimizes seroma formation [6]. The cutaneous surgical incision sites are infiltrated with 1% lidocaine with 1:100,000 epinephrine. The cannula is inserted through two incisions: the first one is made at the mandible angle, and the second one is performed submentally. Every patient underwent tumescent local anesthesia: the tumescent solution was prepared with 25 mL of 2% lidocaine, 8 mEq of sodium bicarbonate, and 1 mL of epinephrine (1 mg/1 mL) in 1000 mL of 0.9% saline solution [7–9]. Overall, 120 mL were introduced. After the TLA infusion, the first incision was made 20 min later to allow epinephrine and lidocaine to have their effect. We used a thin short 3 mm cannula and adopted a “crisscross” pattern of aspiration. This pattern allows the reduction of the degree of postoperative contour irregularities and the appearance of linear cannula lines by breaking the fibrous septae at the level of the subcutaneous fat. In this phase aspiration was not performed. Subsequently, liposuction is carried out with a 3 mm cannula at the level of the deep layer of the aspirated subcutaneous fat leaving a fatty layer of at least 5 mm from the dermis. Eventually, we use the FaceTite cannula (Fig. 1). The area to be treated must be covered with gel. We set 38°C as cutoff for skin temperature, 9-12 Kilojoules, and 20 Watts. The achievement of the temperature and kilojoules cut-off for each individual area gives the end point of the treated area with the radio frequency. After the treatment, the cutaneous incisions are sutured with 5/0 Nylon thread. On average, between 10 and 40 cc of fat per patient is aspirated with the internal cannula. The goal of deeper heating was to obtain contraction of fibrous septae and generate punctuate adhesions of the fat/skin complex to the underlying fascia. Besides the neck tightening procedure, buccal fat pad removal is often performed to enhance facial esthetics. The safest approach to access and remove the buccal fat pad is through intraoral access. Before making an incision, it's important to identify the Stenson duct, which is the opening of the parotid gland into the oral cavity. The incision is typically made either superior to the duct in the maxillary vestibule or inferior to the parotid duct, at the level of the first molar. This allows access to the buccal extension of the posterior lobe. The buccal fat pad is then accessed inferior to Stenson’s duct after local anesthesia injection into the buccal mucosa. Stenson’s duct is positively identified, and a shallow horizontal incision (approximately 1.5 cm) is made with the electrocautery in the buccal mucosa, midway between the occlusal plane and Stenson’s duct. Meanwhile, pressure is applied externally to the buccal fat pad to aid in its exposure into the oral cavity. Next, the buccinator muscle is dissected, allowing access to the buccal fat pad. Once the narrow buccal space is accessed, the yellow fat pad is exposed. A gentle teasing technique is employed to remove 3 to 5 cc of the fat pad from the buccal space (Fig. 2). Hemostats are then used to clamp the fat pad at the level of the mucosa, while surgical scissors are utilized to excise the fat pad. Care must be taken not to apply excessive traction during removal and to only resect the portion of fat that passively protrudes into the oral cavity [10]. Finally, the mucosa is reapproximated using single 4.0 interrupted absorbable sutures. It's crucial to maintain gentle handling throughout the procedure to ensure optimal outcomes and minimize the risk of complications [11, 12]. Follow-up was fixed at 1, 3, 6 and 12 months after surgery. Patients who refused to be followed up, pregnant or breastfeeding women, patients with keloid in the face and neck, and patients with previous liposuction procedures or surgery in the neck region within the previous 6 months were excluded from the study.

## Results

**Fig. 3:**
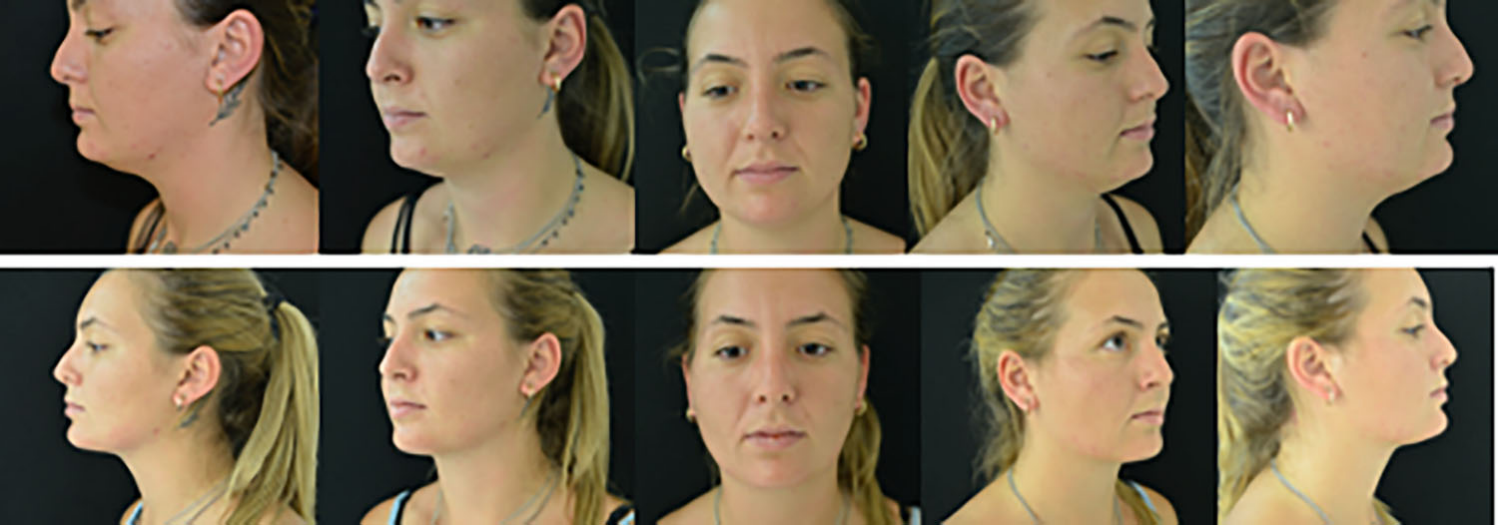
Preoperative view on the top. This patient underwent buccal fat removal surgery and neck radiofrequency with FaceTite. Postoperative view at 6 months follow-up appointment at the bottom

**Fig. 4:**
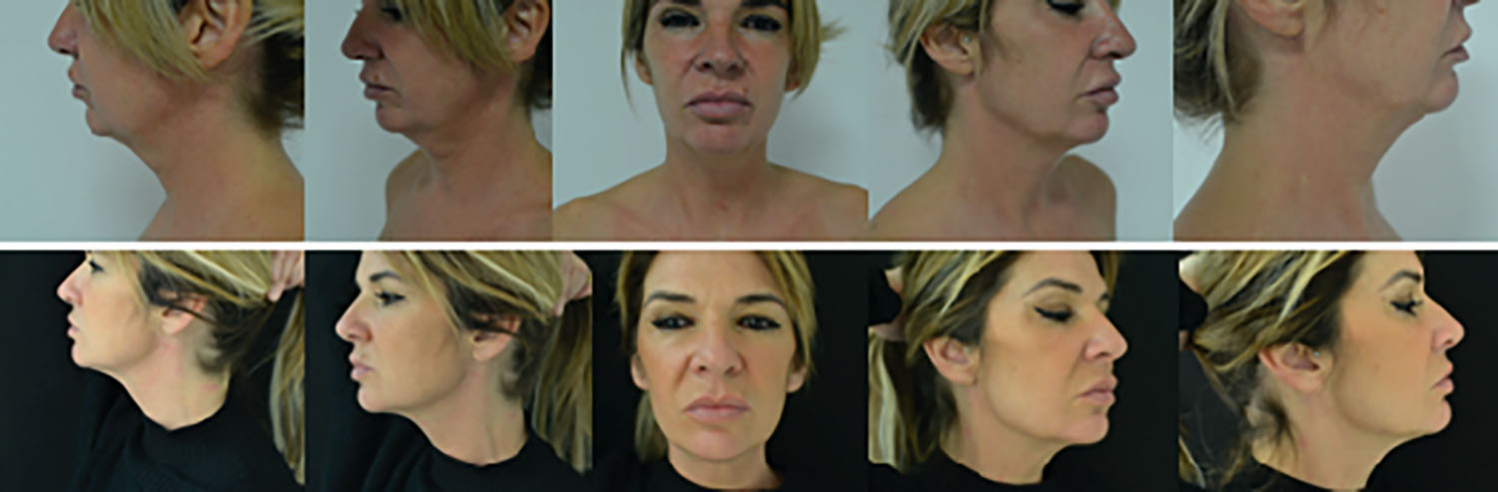
Preoperative view on the top. This patient underwent buccal fat removal surgery and neck radiofrequency with FaceTite. Postoperative view at 6 months follow-up appointment at the bottom

During 8 years, we analyzed 80 patients, 74 women and 6 men, who underwent radiofrequency-assisted liposuction of the neck region and buccal fat pad excision. All procedures were performed using the TLA technique. The median (IQR) age at surgery was 49 years (33–68), and the mean BMI was 29 (range 25–35). The average amount of tumescent solution infiltrated was 120 mL (100–140 mL). No signs of adrenaline or lidocaine toxicity were reported. Our case studies found that the average circumference of the neck at the center of the chin was 19.7 cm before treatment. An average of 25 mL of fat were removed. After treatment, at six months follow-up, we detected 17.4 cm as the average measurement of the submental length (Table 1). We noticed how the measurement was reduced by 75% after the first month, then reached the maximum reduction after six months and eventually stabilized (Figs. 3 and 4). The post-treatment outcomes were juxtaposed with those prior to surgery to assess the extent of improvement subsequent to treatment: among the cohort, 72 patients expressed extreme satisfaction, 7 patients indicated high satisfaction, and 1 patient reported satisfaction; none conveyed slight satisfaction or dissatisfaction (Table 2). Employing the surgeon's four-point scale to gauge overall enhancement, no patient experienced deterioration or remained unchanged, while 4 patients exhibited mild improvement, and 76 patients demonstrated substantial improvement. A comparison between preoperative and postoperative satisfaction was obtained by a satisfaction questionnaire administered to patients. We reported an average satisfaction rate of 22% before treatment, a value of 94% at the sixth month of follow-up and a percentage increase between the two values of 72%. Among the major complications associated with liposuction, it is possible to find in literature hyperesthesia, pain, skin hyperpigmentation, hematoma formation, seroma formation, infections, chronic swelling and skin slough [13, 14]. Unattractive access scars, a lumpy or irregular skin surface, and residual skin laxity can also occur. Furthermore, the passage of the cannula in a plane that is too superficial and, therefore very close to the skin can cause dermal burn or depression [15]. We reported 4 cases (5%) of hyperesthesia which resolved in the first six months. No hematoma, skin slough, chronic swelling, or pain cases were reported. No patients presented seroma formation nor fat necrosis. Six patients (7,5%) noted residual skin laxity which required a revision one year after treatment (Table 3).

**Table 1:** Pre- and post- operative data

Average circumference of the neck before treatment	19.7 cm
Average circumference of the neck after treatment	17.4 cm
Average fat removed	25 mL

**Table 2:** Five-point scale questionnaire results

Number of patients	Five-point scale questionnaire results
72	4 (extreme satisfaction)
7	3 (very high satisfaction)
1	2 (satisfaction)
0	1 (slight satisfaction)
0	0 (dissatisfaction)

**Table 3:** Complications

Complications	Number of patients	Percentage
Hyperesthesia	4	5%
Hematoma	0	0%
Seroma	0	0%
Fat necrosis	0	0%
Skin slough	0	0%
Swelling	0	0%
Pain	0	0%
Unattractive access scars	0	0%

## Discussion

The esthetics of the face have long held a significant role in interpersonal interactions and social dynamics. In contemporary society, the ideals of beauty increasingly advocate for a well-defined facial profile free from imperfections and adiposity, with a pronounced emphasis on achieving a youthful appearance. In this paper, we present our findings from treating 80 patients over an 8-year period, utilizing a combination of neck radiofrequency and buccal fat pad surgical excision. Our objective is to provide patients with results that align harmoniously with their desires and current esthetic standards. Neck lifting and cheek region remodeling represent intricate procedures fraught with complexities. Patient expectations are often elevated, as they seek substantial anti-aging effects and improvements in skin quality. The neck, in particular, is prone to premature aging and is a common area of concern for individuals seeking rejuvenation options [16, 17] and also the mid one third of the face holds a pivotal role in lateral facial profile, significantly influencing one's perceived image [4]. By employing a combination of these two techniques, conducted under local anesthesia and deemed minimally invasive, we have achieved in our study exceptional outcomes in redefining facial contours and neck profile without leaving conspicuous scars or encountering major complications such as bleeding, hematoma, or wound dehiscence, which are inherent risks associated with traditional surgical lifting methods. Historically, non-invasive cervical rejuvenation has been pursued through various methods including chemical ablation, light- or laser-based technologies, ultra-sonic energy, or transcutaneous radiofrequency devices [18]. While each approach has its merits, outcomes often fall short of meeting patient expectations, necessitating multiple procedures or posing risks of significant morbidity. Furthermore, these techniques frequently overlook subdermal adipose tissue, limiting their efficacy for many patients seeking rejuvenation. Another emerging minimally invasive technique gaining popularity involves the use of bioabsorbable threads [19, 20]. This method entails the subcutaneous insertion of threads beneath the skin via needles or cannulas, utilizing barbs to lift, suspend, and support sagging tissue. Critical considerations with this approach include interpersonal variability in thread reabsorption time, typically ranging between 9-12 months, as well as potential complications necessitating urgent attention such as hemorrhage, thread rupture, visibility or palpability, extrusion, infection, and sialocele formation [20]. In our practice, we have employed the FaceTite percutaneous radiofrequency technology, which facilitates simultaneous ablation of subcutaneous fat and tightening of overlying skin. This innovative approach delivers energy directly into the subdermal space, targeting the upper dermal collagen network, deeper fascial layers, and fibrofatty septum [16]. Reported outcomes indicate significant skin tightening and fat reduction, surpassing those achievable with other minimally invasive energy-based technologies. Overall, our experience underscores the efficacy and safety of these advanced techniques in achieving desirable esthetic outcomes while minimizing risks and downtime for patients seeking facial rejuvenation. The surgical procedure of neck lifting stands as a gold standard for addressing skin laxity, subplatysmal fat removal, and reducing platysma laxity. Authors, such as Zins, Guyuron and Knize, described the anterior approach to the neck through a submental incision only [21–23]. This procedure has the added benefit of addressing the subplatysmal plane including subplatysmal fat, and even submandibular gland. Typically performed under general anesthesia, this procedure entails the creation of visible scars and carries the potential for various complications, which can evoke significant concern among patients. In a study conducted by Gordon et al. [24], the authors scrutinized the common complications associated with this procedure. Postoperative hematoma, for instance, is reported to occur in up to 15% of rhytidectomy patients, necessitating immediate attention to prevent complications such as delayed recovery, skin loss, potential airway compromise, and subsequent issues related to pigmentary changes and contour deformities arising from subcutaneous scarring. Infection (0.2–0.6%), seroma formation, skin loss (3–5%), hypertrophic scars, ear deformity, redundant skin, and nerve injury (1.5%) are among the reported complications associated with neck lifting procedures. Furthermore, the authors have detailed complications arising from non-surgical neck lift approaches, emphasizing that transcutaneous energy-based devices, which generate sufficient heat to induce thermal breakdown of collagen fibers, may inadvertently lead to overheating, uneven subcutaneous fat loss, and the formation of scars between the skin and muscle, resulting in neck irregularities. Addressing such issues may necessitate secondary procedures such as microfat injection to restore natural anatomy. In our assessment, FaceTite, with its consistent and controlled thermal effect on the treated area via percutaneous access, offers a predictable and safe means of achieving skin retraction through a minimally invasive approach with a low complication rate. By concentrating radiofrequency (RF) energy between two electrodes—an internal one at the subcutaneous fat level and a larger external one in contact with the skin—selective treatment of specific body areas is facilitated. Notably, higher temperatures are attained in the inner layer while maintaining lower temperatures on the skin surface, thanks to the presence of the outer electrode. This technique enables coagulation of subcutaneous fat and induces scar tissue formation within the fibroseptal network, thereby promoting skin contraction and mitigating the risk of skin burns. Moreover, the device offers precise control over the amount of energy delivered to each treatment area and monitors skin surface temperature in real-time. An acoustic alarm alerts the operator when the pre-set temperature threshold is reached, allowing for timely cessation of treatment on one area before proceeding to the next. While previous studies have used subjective endpoints such as lack of resistance, palpable warmth, and mild erythema to gauge treatment efficacy, we recognize the potential for variability in treatment outcomes based on these criteria [15]. We contend that FaceTite, given its reliance on operator-dependent parameters such as temperature and energy output specific to each treated area, minimizes the likelihood of procedural errors and ensures consistent treatment outcomes across different operators. In our study, we also addressed the buccal fat pad, alternatively known as Bichat’s fat pad, a significant anatomical structure that significantly influences facial esthetics and aids in the mobility of facial muscles. Numerous studies have explored the versatility of the buccal fat pad, particularly in the realms of oral defect reconstruction and trauma management, where it can serve as a pedicled autogenous graft for repairing oroantral fistulas [25]. However, beyond its functional role, the buccal fat pad holds considerable importance in facial esthetics and can be strategically modified to refine facial contours and enhance overall appearance [26]. The removal of the buccal fat pad contributes to the enhancement of the middle and lower thirds of the face, accentuating the malar prominence and fostering a sculpted facial appearance [26]. Imaging studies reveal a significant growth of the buccal fat pad from ages 10 to 20, with volumes increasing from 4000 to 8000 cubic millimeters, followed by a gradual decline over the subsequent 30 years to an average volume of 7000 cubic millimeters [27]. Described as a round, encapsulated, biconvex structure situated in the buccal space, the buccal fat pad resides between the anterior margin of the masseter and the buccinator, with the parotid gland posteriorly positioned. The mean volumetric variation is noted to range from 7.8 to 11.2 milliliters for males and 7.2 to 10.8 milliliters for females, accompanied by a mean thickness of 6 millimeters. It maintains proximity to the facial nerve, parotid duct, and masticatory muscles [25]. The body of the buccal fat pad is compartmentalized into three lobes: anterior, intermediate, and posterior, with the posterior lobe characterized by four extensions: buccal process, pterygopalatine process, pterygoid process, and temporal process [28]. The posterior lobe, synonymous with the body, constitutes the most substantial portion of the buccal fat pad and is confined within the masticatory space. Approximately 50% of the buccal fat pad volume is attributed to the buccal extension and body [29]. The buccal extension, positioned inferior to Stenon’s Duct, represents the most superficial process and we typically remove it during buccal fat pad resection. Ideal candidates for this procedure exhibit strong malar bones, albeit concealed by prominent cheeks, resulting in an appearance of excessive facial roundness and a heavier facial esthetic. However, caution is warranted, as the procedure is contraindicated in individuals with hypoplastic malar bones, as overcorrection may yield unfavorable outcomes. We employ the technique outlined by Matarasso for buccal fat pad removal, utilizing an intraoral approach under local anesthesia [10]. After identifying Stenon’s duct, we create a 1.5 cm horizontal incision approximately 1 cm below this structure. Concurrently, pressure is applied externally with the opposite hand to facilitate exposure of the buccal fat pad into the oral cavity. Hemostats are then used to bluntly dissect through the buccinator muscle, gaining access to the buccal fat pad. Following exposure, we excise 3 to 5 cc of fat from the pad, considering the individual proportions of the patient's face and the desired outcome. Thorough hemostasis is achieved, and the mucosa is sutured using two single 4.0 interrupted absorbable stitches. Care is taken to avoid excessive traction during removal and to resect only the fat portion that passively protrudes into the oral cavity [11]. Complications associated with buccal fat pad removal are rare, with reported rates ranging from 8.45% to 18%. These may include hematoma, injury to the parotid gland duct, trismus, neuromotor deficits, and infection [30]. In our case history, on over of 80 surgeries performed, there are no reported cases of complications that required further conservative or surgical treatments. Knowledge of the buccal fat pad and its related vital structures combined with the correct surgical technique are fundamental to mitigating complications associated with its reduction. Alcantara et al. described two cases of complications following BFP reduction [31]. One case involved the accumulation of saliva in the buccal mucosa due to obstruction of the Stenson duct resulting from iatrogenic damage. Conservative therapy and multiple drainages of the right buccal mucosa were employed, resulting in the re-establishment of adequate salivary drainage and discharge of the patient.

## Conclusion

Many of our surgeries have been conducted primarily for cosmetic enhancement. Whether undertaken to refine the neck and facial contours through procedures such as neck radiofrequency treatment or surgical reduction of the Bichat Buccal fat pad, these interventions offer a safe and beneficial option in terms of achieving favorable esthetic outcomes, minimizing complication rates, and facilitating post-operative recovery. Moreover, such procedures have the potential to enhance the patient's body image and contribute to improvements in health-related quality of life.
